# 1-Acetyl-5-(4-fluoro­phen­yl)-2-sulfanyl­ideneimidazolidin-4-one

**DOI:** 10.1107/S1600536813028560

**Published:** 2013-10-23

**Authors:** Soh-ichi Kitoh, Yijing Feng, Shuhei Fujinami, Masaki Ichitani, Mitsunori Honda, Ko-Ki Kunimoto

**Affiliations:** a Division of Material Sciences, Graduate School of Natural Science and Technology, Kanazawa University, Kakuma-machi, Kanazawa 920-1192, Japan

## Abstract

In the title compound, C_11_H_9_FN_2_O_2_S, the 2-sulfanylideneimidazolidin-4-one moiety is essentially planar, with a maximum deviation of 0.0183 (14) Å. The mean plane of this moiety is approximately coplanar with the attached acetyl group and perpendicular to the benzene ring, making dihedral angles of 9.70 (14) and 86.70 (6)°, respectively. In the crystal, mol­ecules are linked by N—H⋯O hydrogen bonds between the amide NH and acetyl C=O groups, forming a *C*(6) chain along the *a-*axis direction.

## Related literature

For applications and the biological activity of 2-sulfanylideneimidazolidin-4-ones, see: Marton *et al.* (1993 ▶). For the crystal structures of related compounds, see: Casas *et al.* (1998 ▶); Sulbaran *et al.* (2007 ▶); Taniguchi *et al.* (2009 ▶). For a description of the Cambridge Structural Database, see: Allen (2002 ▶). For hydrogen-bond motifs, see: Etter (1990 ▶). For the synthetic procedure, see: Schlack & Kumpf (1926 ▶).
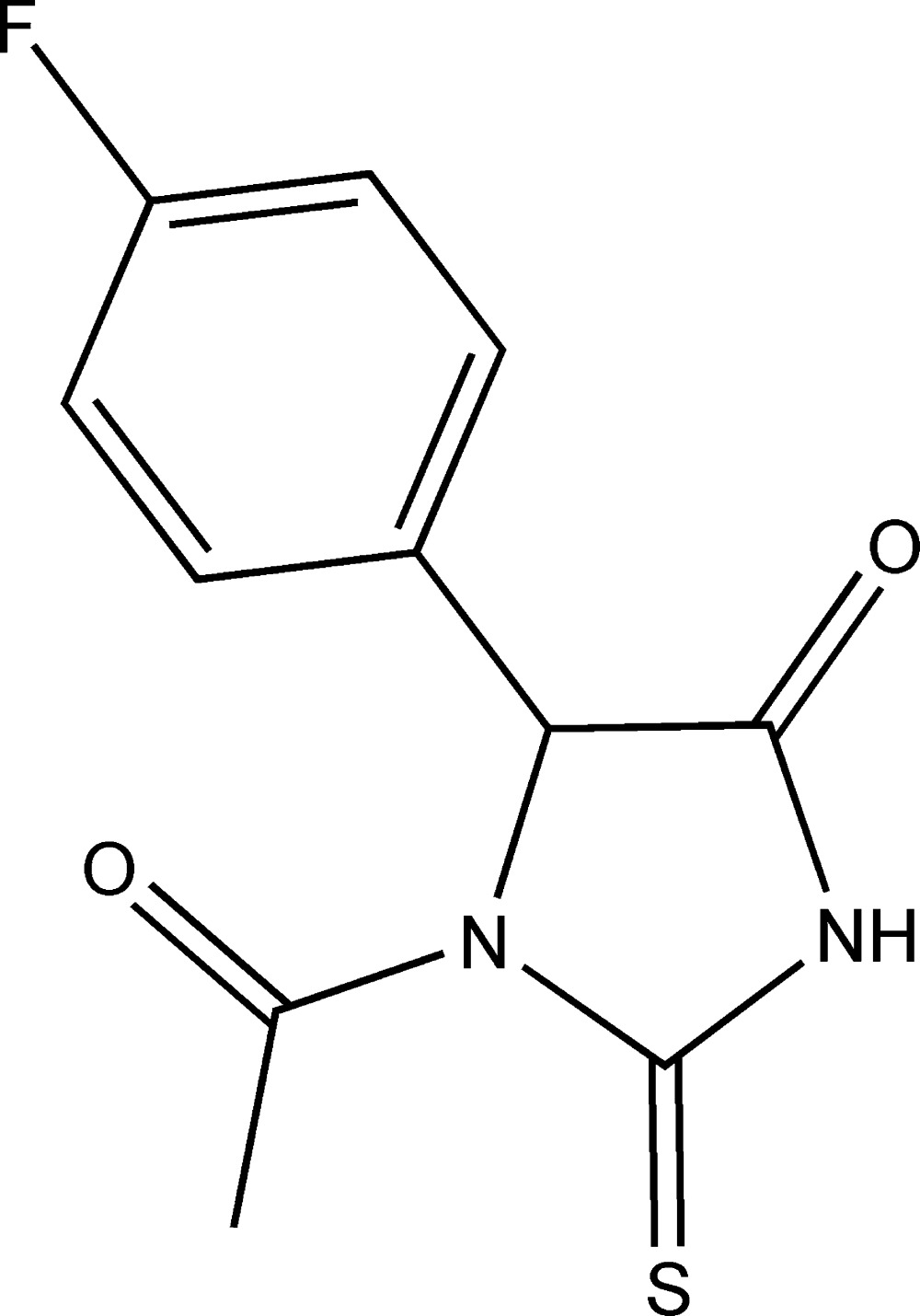


## Experimental

### 

#### Crystal data

C_11_H_9_FN_2_O_2_S
*M* *_r_* = 252.27
Monoclinic, 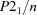

*a* = 7.1327 (9) Å
*b* = 23.852 (3) Å
*c* = 7.3437 (10) Å
β = 113.541 (3)°
*V* = 1145.4 (3) Å^3^
*Z* = 4
Mo *K*α radiation
μ = 0.29 mm^−1^
*T* = 123 K
0.30 × 0.10 × 0.08 mm

#### Data collection

Rigaku/MSC Mercury CCD diffractometer
Absorption correction: multi-scan (*REQAB*; Rigaku, 1998 ▶) *T*_min_ = 0.829, *T*_max_ = 0.977
12234 measured reflections
2612 independent reflections
2418 reflections with *F* ^2^ > 2σ(*F* ^2^)
*R*_int_ = 0.024

#### Refinement

*R*[*F* ^2^ > 2σ(*F* ^2^)] = 0.031
*wR*(*F* ^2^) = 0.082
*S* = 1.06
2612 reflections
159 parameters
H atoms treated by a mixture of independent and constrained refinement
Δρ_max_ = 0.32 e Å^−3^
Δρ_min_ = −0.23 e Å^−3^

### 

Data collection: *CrystalClear* (Rigaku, 2006 ▶); cell refinement: *CrystalClear*; data reduction: *CrystalClear*; program(s) used to solve structure: *SIR2008* in *Il Milione* (Burla *et al.*, 2007 ▶); program(s) used to refine structure: *SHELXL97* (Sheldrick, 2008 ▶); molecular graphics: *ORTEP-3 for Windows* (Farrugia, 2012 ▶) and *Mercury* (Macrae *et al.*, 2006 ▶); software used to prepare material for publication: *CrystalStructure* (Rigaku, 2010 ▶).

## Supplementary Material

Crystal structure: contains datablock(s) General, I. DOI: 10.1107/S1600536813028560/is5315sup1.cif

Structure factors: contains datablock(s) I. DOI: 10.1107/S1600536813028560/is5315Isup2.hkl

Supplementary material file. DOI: 10.1107/S1600536813028560/is5315Isup3.cml

Additional supplementary materials:  crystallographic information; 3D view; checkCIF report

## Figures and Tables

**Table 1:** Hydrogen-bond geometry (Å, °)

*D*—H⋯*A*	*D*—H	H⋯*A*	*D*⋯*A*	*D*—H⋯*A*
N2—H2⋯O2^i^	0.84 (2)	1.96 (2)	2.7836 (16)	167 (2)

Symmetry code: (i) 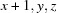.

**Fig. 1.**
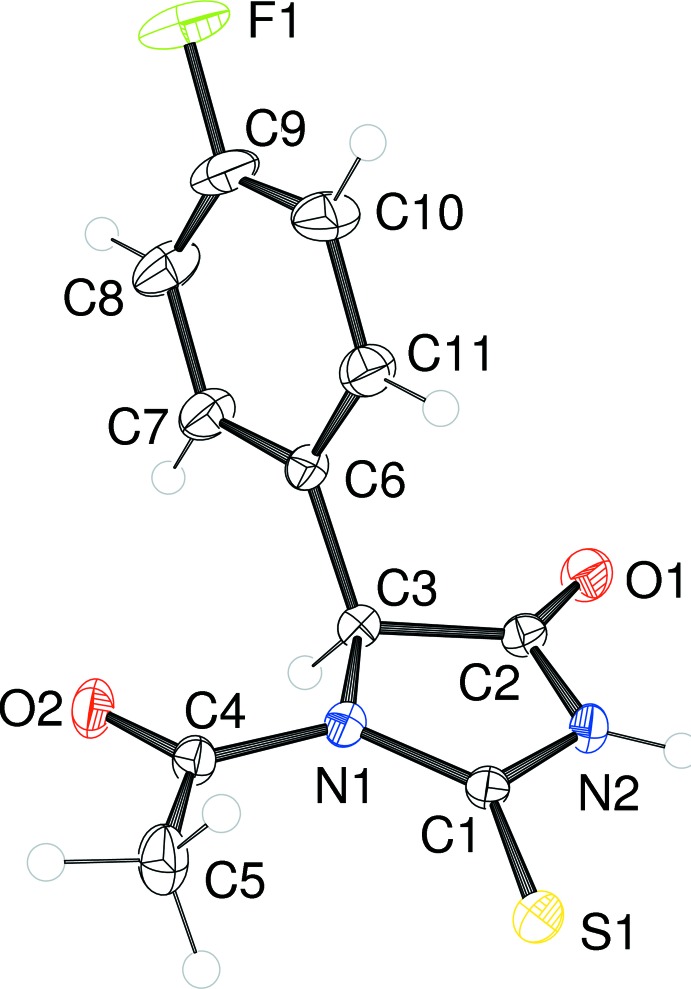
The molecular structure of the title compound, with the atom-numbering scheme. Displacement ellipsoids are drawn at the 50% probability level.

**Fig. 2.**
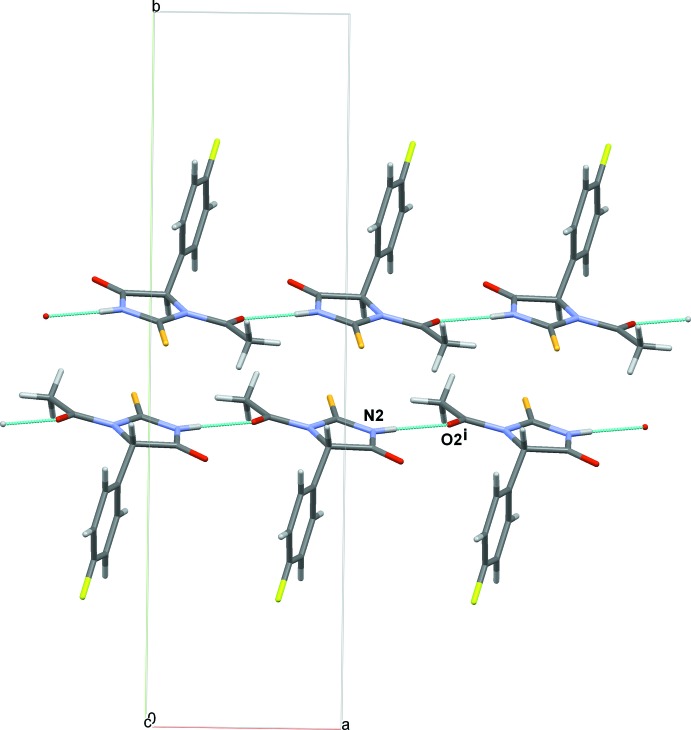
A partial packing diagram of the title compound, viewed down the c axis. Hydrogen bonds are shown as dashed cyan lines (see Table 1 for details).

## supplementary crystallographic information

### 1. Comment

2-Sulfanylideneimidazolidin-4-one (2-thiohydantoin) derivatives are useful synthetic intermediates in a wide range of applications, such as therapeutics, fungicides and herbicides (Marton *et al.*, 1993). We have been studying crystal structures and hydrogen-bonding patterns of the polymorphic forms of 2-thiohydantoin derivatives. The Cambridge Structural Database survey (Ver. 5.34; Allen, 2002) indicates that 1-acetyl-2-thiohydantoins with an unsubstituted N atom show three types of N—H···O hydrogen-bonding patterns: (i) the amide NH and the acetyl C═O groups form a chain with a *C*(6) graph-set motif (Etter *et al.*, 1990) [triclinic polymorph of 1-acetyl-2-thiohydantoin (NIFHIT01) (Taniguchi *et al.*, 2009) and two other derivatives (KABRIQ and KOMGUO)]; (ii) the amide NH and the amide C═ O groups form a chain with *C*(4) [monoclinic polymorph of 1-acetyl-2-thiohydantoin (NIFHIT) (Casas *et al.*, 1998) and one other derivative (DOKXUX)]; (iii) the amide NH and the amide C═O groups form a ring with *R*^2^_2_(8) [1-acetyl-5-methyl-2-thiohydantoin (DIKWAW) (Sulbaran *et al.*, 2007)]. As an extension of our research, we report on the crystal structure of the title compound, C_11_H_9_FN_2_O_2_S.

In the title molecule (Fig. 1), the bond lengths and angles are normal and comparable to those observed in the reported 1-acetyl-2-thiohydantoins with an unsubstituted N atom. The 2-thiohydantoin moiety (N1/C1/S1/N2/C2/O1/C3) is essentially planar, with maximum deviations of 0.0183 (14) Å for C3 atom and -0.0138 (13) Å for N1 atom. The acetyl group (C4/O2/C5) is almost coplanar with the 2-thiohydantoin moiety, and the dihedral angle between the acetyl group and the 2-thiohydantoin moiety is 9.70 (14)°.

In the crystal structure (Fig. 2), the molecules are linked by an N—H···O hydrogen bond between the amide NH and acetyl C═O groups, forming a infinite one-dimensional chain along the *a* axis, with a *C*(6) graph-set motif (Table 1).

### 2. Experimental

The title compound was synthesized using a slight modification of a reported method (Schlack & Kumpf, 1926). 4-Fluorophenylglycine (0.507 g, 3.00 mmol) was allowed to react with a mixture of ammonium thiocyanate (0.234 g, 3.07 mmol), acetic anhydride (10 ml), and acetic acid (2 ml) at 100 °C for 1 h. A white precipitate was obtained by adding 25 ml distilled water and subsequent cooling the solution in a refrigerator. The crude product was purified by recrystallization from an ethanol solution (yield: 47%). Single crystals suitable for X-ray diffraction were obtained from the ethanol solution.

### 3. Refinement

The N-bound H atom was located in a difference map and refined freely [N2—H2 = 0.84 (2) Å]. The remaining H atoms were positioned geometrically (C—H = 0.95, 0.98 or 1.00 Å) and refined using a riding model, with *U*_iso_(H) = 1.2 *U*_eq_(C). A rotating group model was applied to the methyl group.

### Crystal data

**Table:**

C_11_H_9_FN_2_O_2_S	*F*(000) = 520
*M**_r_* = 252.27	*D*_x_ = 1.463 Mg m^−^^3^
Monoclinic, *P*2_1_/*n*	Mo *K*α radiation, λ = 0.71070 Å
Hall symbol: -P 2yn	Cell parameters from 4829 reflections
*a* = 7.1327 (9) Å	θ = 3.0–27.5°
*b* = 23.852 (3) Å	µ = 0.29 mm^−^^1^
*c* = 7.3437 (10) Å	*T* = 123 K
β = 113.541 (3)°	Prism, colorless
*V* = 1145.4 (3) Å^3^	0.30 × 0.10 × 0.08 mm
*Z* = 4	

### Data collection

**Table:**

Rigaku/MSC Mercury CCD diffractometer	2418 reflections with *F*^2^ > 2σ(*F*^2^)
Detector resolution: 7.314 pixels mm^-1^	*R*_int_ = 0.024
ω scans	θ_max_ = 27.5°, θ_min_ = 3.4°
Absorption correction: multi-scan (*REQAB*; Rigaku, 1998)	*h* = −9→9
*T*_min_ = 0.829, *T*_max_ = 0.977	*k* = −30→30
12234 measured reflections	*l* = −9→8
2612 independent reflections	

### Refinement

**Table:**

Refinement on *F*^2^	Secondary atom site location: difference Fourier map
*R*[*F*^2^ > 2σ(*F*^2^)] = 0.031	Hydrogen site location: inferred from neighbouring sites
*wR*(*F*^2^) = 0.082	H atoms treated by a mixture of independent and constrained refinement
*S* = 1.06	*w* = 1/[σ^2^(*F*_o_^2^) + (0.0441*P*)^2^ + 0.4044*P*] where *P* = (*F*_o_^2^ + 2*F*_c_^2^)/3
2612 reflections	(Δ/σ)_max_ = 0.001
159 parameters	Δρ_max_ = 0.32 e Å^−^^3^
0 restraints	Δρ_min_ = −0.23 e Å^−^^3^
Primary atom site location: structure-invariant direct methods	

### Special details

**Table:**

Geometry. All e.s.d.'s (except the e.s.d. in the dihedral angle between two l.s. planes) are estimated using the full covariance matrix. The cell e.s.d.'s are taken into account individually in the estimation of e.s.d.'s in distances, angles and torsion angles; correlations between e.s.d.'s in cell parameters are only used when they are defined by crystal symmetry. An approximate (isotropic) treatment of cell e.s.d.'s is used for estimating e.s.d.'s involving l.s. planes.
Refinement. Refinement was performed using all reflections. The weighted *R*-factor (*wR*) and goodness of fit (*S*) are based on *F*^2^. *R*-factor (gt) are based on *F*. The threshold expression of *F*^2^ > 2.0 σ(*F*^2^) is used only for calculating *R*-factor (gt).

### Fractional atomic coordinates and isotropic or equivalent isotropic displacement parameters (Å^2^)

**Table:**

	*x*	*y*	*z*	*U*_iso_*/*U*_eq_	
S1	0.92257 (4)	0.464054 (12)	0.18414 (4)	0.02040 (10)	
F1	0.66717 (16)	0.18005 (4)	0.78913 (15)	0.0472 (3)	
O1	1.28538 (13)	0.37499 (4)	0.83947 (13)	0.0254 (2)	
O2	0.53419 (13)	0.42791 (4)	0.53528 (13)	0.0240 (2)	
N1	0.81733 (14)	0.42098 (4)	0.47872 (14)	0.0166 (2)	
N2	1.14408 (15)	0.41667 (4)	0.53118 (15)	0.0180 (2)	
C1	0.95711 (17)	0.43386 (4)	0.39663 (17)	0.0164 (3)	
C2	1.14023 (17)	0.39352 (5)	0.70131 (17)	0.0186 (3)	
C3	0.91869 (16)	0.39534 (5)	0.67800 (16)	0.0166 (3)	
C4	0.61024 (17)	0.43576 (5)	0.41600 (18)	0.0187 (3)	
C5	0.49412 (19)	0.45857 (6)	0.21277 (19)	0.0265 (3)	
C6	0.84028 (17)	0.33792 (5)	0.69937 (17)	0.0184 (3)	
C7	0.7772 (2)	0.32809 (5)	0.8518 (2)	0.0264 (3)	
C8	0.7187 (3)	0.27457 (6)	0.8832 (2)	0.0338 (4)	
C9	0.7236 (3)	0.23252 (6)	0.7578 (3)	0.0314 (3)	
C10	0.7823 (3)	0.24057 (6)	0.6034 (2)	0.0304 (3)	
C11	0.8411 (2)	0.29430 (5)	0.57419 (19)	0.0249 (3)	
H2	1.252 (3)	0.4205 (7)	0.513 (3)	0.027 (4)*	
H3	0.9063	0.4211	0.7800	0.0199*	
H5A	0.3491	0.4617	0.1885	0.0318*	
H5B	0.5095	0.4333	0.1143	0.0318*	
H5C	0.5474	0.4957	0.2019	0.0318*	
H7	0.7738	0.3581	0.9356	0.0317*	
H8	0.6765	0.2673	0.9884	0.0406*	
H10	0.7828	0.2104	0.5189	0.0364*	
H11	0.8822	0.3012	0.4681	0.0299*	

### Atomic displacement parameters (Å^2^)

**Table:**

	*U*^11^	*U*^22^	*U*^33^	*U*^12^	*U*^13^	*U*^23^
S1	0.02194 (17)	0.02105 (16)	0.02123 (17)	0.00019 (10)	0.01181 (13)	0.00233 (10)
F1	0.0632 (7)	0.0242 (5)	0.0522 (6)	−0.0180 (4)	0.0208 (5)	0.0062 (4)
O1	0.0181 (5)	0.0278 (5)	0.0267 (5)	0.0001 (4)	0.0052 (4)	0.0056 (4)
O2	0.0175 (4)	0.0300 (5)	0.0283 (5)	0.0008 (4)	0.0131 (4)	0.0028 (4)
N1	0.0148 (5)	0.0173 (5)	0.0188 (5)	−0.0003 (4)	0.0078 (4)	0.0014 (4)
N2	0.0141 (5)	0.0196 (5)	0.0223 (5)	−0.0004 (4)	0.0095 (4)	−0.0004 (4)
C1	0.0170 (5)	0.0122 (5)	0.0220 (6)	−0.0019 (4)	0.0099 (5)	−0.0034 (4)
C2	0.0183 (6)	0.0156 (5)	0.0231 (6)	−0.0024 (4)	0.0094 (5)	−0.0016 (4)
C3	0.0162 (6)	0.0163 (6)	0.0179 (6)	−0.0008 (4)	0.0075 (5)	0.0006 (4)
C4	0.0153 (6)	0.0183 (6)	0.0235 (6)	−0.0011 (5)	0.0087 (5)	−0.0018 (5)
C5	0.0174 (6)	0.0386 (8)	0.0229 (6)	0.0029 (5)	0.0072 (5)	0.0036 (5)
C6	0.0151 (5)	0.0180 (6)	0.0218 (6)	−0.0016 (4)	0.0072 (5)	0.0006 (5)
C7	0.0316 (7)	0.0243 (7)	0.0279 (7)	−0.0064 (5)	0.0168 (6)	−0.0020 (5)
C8	0.0424 (8)	0.0321 (8)	0.0323 (7)	−0.0116 (6)	0.0205 (7)	0.0034 (6)
C9	0.0326 (7)	0.0207 (6)	0.0370 (8)	−0.0097 (6)	0.0098 (6)	0.0061 (6)
C10	0.0363 (8)	0.0187 (6)	0.0353 (8)	−0.0058 (6)	0.0135 (6)	−0.0051 (5)
C11	0.0284 (7)	0.0219 (6)	0.0278 (7)	−0.0035 (5)	0.0146 (6)	−0.0022 (5)

### Geometric parameters (Å, º)

**Table:**

S1—C1	1.6454 (13)	C7—C8	1.391 (2)
F1—C9	1.3621 (19)	C8—C9	1.372 (3)
O1—C2	1.2073 (13)	C9—C10	1.370 (3)
O2—C4	1.2150 (19)	C10—C11	1.392 (2)
N1—C1	1.3899 (19)	N2—H2	0.84 (2)
N1—C3	1.4810 (14)	C3—H3	1.000
N1—C4	1.4053 (16)	C5—H5A	0.980
N2—C1	1.3676 (14)	C5—H5B	0.980
N2—C2	1.3762 (18)	C5—H5C	0.980
C2—C3	1.5206 (18)	C7—H7	0.950
C3—C6	1.5111 (18)	C8—H8	0.950
C4—C5	1.4906 (17)	C10—H10	0.950
C6—C7	1.383 (3)	C11—H11	0.950
C6—C11	1.3900 (19)		
			
C1—N1—C3	111.61 (9)	C8—C9—C10	123.48 (15)
C1—N1—C4	130.13 (10)	C9—C10—C11	117.85 (14)
C3—N1—C4	117.51 (11)	C6—C11—C10	120.41 (15)
C1—N2—C2	114.14 (12)	C1—N2—H2	123.1 (10)
S1—C1—N1	130.27 (8)	C2—N2—H2	122.7 (10)
S1—C1—N2	123.36 (11)	N1—C3—H3	109.392
N1—C1—N2	106.37 (11)	C2—C3—H3	109.399
O1—C2—N2	126.05 (13)	C6—C3—H3	109.408
O1—C2—C3	127.51 (13)	C4—C5—H5A	109.473
N2—C2—C3	106.44 (9)	C4—C5—H5B	109.477
N1—C3—C2	101.43 (11)	C4—C5—H5C	109.470
N1—C3—C6	114.97 (9)	H5A—C5—H5B	109.474
C2—C3—C6	111.93 (10)	H5A—C5—H5C	109.461
O2—C4—N1	116.01 (10)	H5B—C5—H5C	109.473
O2—C4—C5	123.27 (12)	C6—C7—H7	119.804
N1—C4—C5	120.71 (13)	C8—C7—H7	119.807
C3—C6—C7	119.42 (11)	C7—C8—H8	120.984
C3—C6—C11	120.67 (13)	C9—C8—H8	120.972
C7—C6—C11	119.82 (12)	C9—C10—H10	121.071
C6—C7—C8	120.39 (14)	C11—C10—H10	121.082
C7—C8—C9	118.04 (17)	C6—C11—H11	119.797
F1—C9—C8	118.03 (16)	C10—C11—H11	119.789
F1—C9—C10	118.50 (14)		
			
C1—N1—C3—C2	−1.25 (11)	O1—C2—C3—C6	−55.41 (16)
C1—N1—C3—C6	−122.21 (10)	N2—C2—C3—N1	0.71 (11)
C3—N1—C1—S1	−178.11 (9)	N2—C2—C3—C6	123.78 (9)
C3—N1—C1—N2	1.32 (11)	N1—C3—C6—C7	−126.90 (11)
C1—N1—C4—O2	−166.54 (10)	N1—C3—C6—C11	56.50 (14)
C1—N1—C4—C5	14.60 (17)	C2—C3—C6—C7	118.06 (11)
C4—N1—C1—S1	−8.49 (18)	C2—C3—C6—C11	−58.54 (12)
C4—N1—C1—N2	170.93 (10)	C3—C6—C7—C8	−175.27 (9)
C3—N1—C4—O2	2.57 (15)	C3—C6—C11—C10	175.46 (9)
C3—N1—C4—C5	−176.29 (9)	C7—C6—C11—C10	−1.12 (16)
C4—N1—C3—C2	−172.31 (9)	C11—C6—C7—C8	1.35 (17)
C4—N1—C3—C6	66.73 (13)	C6—C7—C8—C9	−0.67 (18)
C1—N2—C2—O1	179.24 (10)	C7—C8—C9—F1	179.71 (11)
C1—N2—C2—C3	0.04 (12)	C7—C8—C9—C10	−0.3 (2)
C2—N2—C1—S1	178.64 (9)	F1—C9—C10—C11	−179.49 (10)
C2—N2—C1—N1	−0.83 (12)	C8—C9—C10—C11	0.5 (2)
O1—C2—C3—N1	−178.48 (12)	C9—C10—C11—C6	0.21 (18)

### Hydrogen-bond geometry (Å, º)

**Table:**

*D*—H···*A*	*D*—H	H···*A*	*D*···*A*	*D*—H···*A*
N2—H2···O2^i^	0.84 (2)	1.96 (2)	2.7836 (16)	167 (2)

Symmetry code: (i) *x*+1, *y*, *z*.
